# Allergies and risk of colorectal cancer: a systematic review and meta-analysis of observational studies

**DOI:** 10.18632/oncotarget.14599

**Published:** 2017-01-11

**Authors:** Jianrong Ye, Ailaiti Talaiti, Yan Ma, Qin Zhang, Long Ma, Hong Zheng

**Affiliations:** ^1^ Department of Anesthesiology, First Affiliated Hospital of Xinjiang Medical University, Urumqi, Xinjiang, China

**Keywords:** allergy, colorectal cancer, observational study, meta-analysis

## Abstract

A history of allergy or allergic condition has been reported to be associated with reduced risk of some types of malignancies. However, the understanding of this association for colorectal cancer (CRC) is controversial. We conducted a meta-analysis of CRC risk in individuals who had history of allergy compared to those without the history of allergic condition. Pumbed and Embase databases were searched for relevant studies. The adjusted relative risk (RR) and 95% confidence interval (CI) were pooled using the random-effects model. Nine studies, including 775, 178 individuals, were eligible for inclusion. The pooled estimate showed no significant association between history of allergy and CRC risk (adjusted RR 1.01, 95 % CI 0.88–1.17). Subgroup analyses confirmed the neutral association stratified by tumor location (colon: *n* = 6 studies; adjusted RR 1.01, 95 % CI 0.81–1.25; rectum: *n* = 6 studies; adjusted RR 0.94, 95% CI 0.77–1.15; colorectum: *n* = 3 studies; adjusted RR 0.92, 95 % CI 0.70 to 1.21), sex (male: *n* = 4 studies; adjusted RR 0.93, 95 % CI 0.81–1.07; female: *n* = 6 studies; adjusted RR 0.94, 95 % CI 0.80–1.09) or by allery type (asthma: *n* = 5 studies; adjusted RR 1.16, 95 % CI 0.96–1.42; hay fever: *n* = 4 studies; adjusted RR 0.93, 95 % CI 0.86–1.03). Meta-analysis of existing evidence provides a neutral association between allergies and CRC risk. Future well-designed prospective cohort studies should be conducted to better understand this association.

## INTRODUCTION

Colorectal cancer (CRC) represents the third most common cancer and the fourth cause of cancer death worldwide, with an estimated 1.4 million new colorectal cancer cases and 693,900 deaths in 2012 globally [1]. Allergies, known as asthma, hay fever/allergic rhinitis and other allergy-related conditions have been reported to be linked with risk of various cancer types [2–8], indicating a potential preventive effect of allergic conditions against CRC [9–10]. Studies have suggested the potential protective effect of allergy on cancer development through activating IgE-mediated immune reactions for cancer cell, inducing the protective role of ACCs for various tumors, such as lung cancer and brain cancer [2, 8, 11].

Accumulating evidence suggests a pivotal role of allergic conditions in modulation of immune function [12, 13]. We have long recognized the important roles of host immunity and inflammation in regulating tumour evolution [14–18]. Local immunity of tumour microenvironment may obliterate cancerous cells, promoting or preventing their tumourigenic potential, thus determining the fate of emerging tumour. However, despite vast evidence for the role of allergic conditions in immunity and the role of immunity in tumour development, no consensus among studies has yet been reached whether there was association between allergies and CRC risk. The purpose of this study was to conduct a meta-analysis of CRC risk in individuals who had history of allergy compared to those without the history of allergic condition.

## RESULTS

We performed this meta-analysis in accordance with the Preferred Reporting Items for Systematic reviews and Meta-Analyses (PRISMA) guidelines [19] (Supplementary Table S4).

### Search and selection of studies

**Figure 1:**
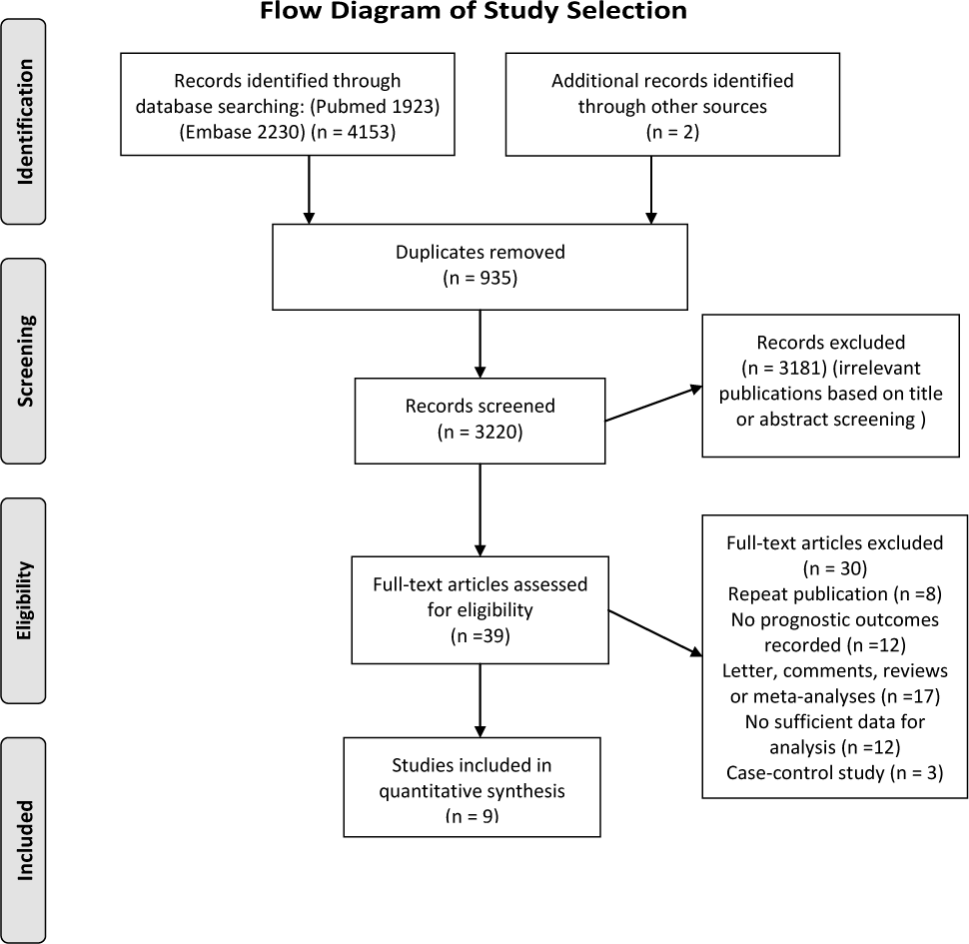
Flow diagram of the study selection

We retrieved 3,220 unique citations in the initial literature search and 39 potentially relevant studies for full-text review. After removing 30 studies, a total of 9 cohort studies met our inclusion criteria and were involved in the meta-analysis [20–28] (Figure 1).

### Study characteristics

The baseline characteristics of the identified studies are shown in Table 1. A total of 775,178 participants were included in this study with a median sample size of 77,952 (range, 3,308 to 199,112). The median follow-up period was 10.9 (range 8 to 18) years. All of the included studies were published between 1993 and 2015 in English peer-reviewed journals.

**Table 1:** Baseline characteristics of the included studies

Study	Year	Region	Inclusion period	Study name	Single or multicenter	Study design	Sample size (Exposure No.)	Mean or median age (ys)	Male Sex (%)	Type of allergic conditions	Exposure assessment method	Measure ofassociations	Outcome assessment registry	Years of follow-up	Adjusted variables
Tambe et al.	2015	USA	1993-1996	Multiethnic Cohort Study of Diet and Cancer	M	Population based prospective cohort study	199112 (51973)	Mean (E/NE): 59.38/60.81	89496 (44.95)	Asthma, hay fever, or allergy	A detailed, 26-page self-administeredquestionnaire	RR	Cohort linkage withthe Surveillance, Epidemiology, and End Results (SEER) registries	17	Age at entry into the cohort, sex, and ethnicity, smoking status,pack-years, educational level,body mass index,aspirin usage,vigorous physical activity, alcohol intake, saturated fat intake, nonsaturated fat intake, dietary fiber intake,energy intake,andafamily history of CRC
Skaaby et al.	2014	Denmark	1976-2006	5 Danish studies including Monica1, Allergy98, Inter99, Health2006 and 1936-cohort.	M	Population based prospective cohort study	14849 (3994)	Mean Monica1:45; Allergy98: 40; Inter99: 46.1; Health2006: 49.0; 1936-cohort: 40.4	7155 (48.18)	Atopy	Questionnaires, physical examinations, andblood tests	HR	Danish Cancer Register	11.8	Study,sex,education, activity, smoking habits, alcohol intake, and BMI
Jacobs et al.	2013	USA	1992-1993	CPS-II Nutrition Cohort	M	Population based prospective cohort study	174917 (26238)	>40	26208 (52.9)	Asthma, hay fever	A mailed self-administered questionnaire	RR	Medical records, or through linkagewith state cancer registries; linkage with the National Death Index	10	Age, sex, race, education, BMI, physical activity, smoking, aspirin use, and history ofcolorectal endoscopy
Chae et al.	2012	USA	1988-1994	NHANES III Female Cohort	M	Population based retrospective cohort study	4600 (2331)	Mean 59.56	0	RC: symptoms of allergic rhinitis or conjunctivitis without wheezing; WZ: wheezing	Self-administered questionnaire	OR	Self-reported physician diagnosed canceras well as a cancer diagnosis listed on the records of hospital ornursing home, or on death certificate if hospital record of cancerwas not available.	NR	Age, race, education,income, asthma, COPD, C-reactive protein, obesity, smoking,alcohol drinking, physical inactivity, and menopausal status.
Prizment et al.	2007	USA	1997-2004	Iowa Women’s Health Study	S	Populationbased prospective cohort study	21,292 (6,765)	Mean 72.1	0	Asthma, hay fever, eczema or allergy of the skin, or other allergic conditions	Five self-administered questionnaire	HR	Annual linkage to the StateHealth Registry of Iowa, part of the Surveillance, Epidemiology, and End Results Program.	8	Age, pack-years, total energy intake, calcium, red meat, and multivitamin use in 1986, BMI in 1997, and diabetes and HRT use before 1997
Talbot-Smith et al.	2002	Australia	1981-1999	The 1981 Busselton Health Survey	M	Community-based, prospective study	3308 (1343)	Mean male: 50.7; female: 50	1522(46.01)	Asthma, hay fever, atopy	A history of physician-diagnosed based on a questionnaires	HR	Linkage tothe West Australian Cancer Registry	18	Age, smoking status, and body mass index
Hemminki et al.	2014	Germany(sweden)	1964-2010	Swedish nationwide health care cohort	M	Nationwide populationbased prospective cohort study	138723	Mean 24	70521 (50.84)	Hay fever, allergic rhinitis	Three Swedish health care databases(Hospital Discharge Register,Outpatient Registry,Primary Health CareRegistry)	SIR	Swedish Cancer Registry	8	None
Vesterinen et al.	1993	Finland	1970-1985	FInland nationwide cohortstudy	M	Nationwide populationbased prospective cohort study	77952	35-84	35126 (45.06)	Asthma	Nationwide Social Insurance Institution register	SIR	Finnish Cancer Registry		None
Ji et al.	2009	Swedish	1965-2004	Swedish Hospital Discharge Register cohort	M	Nationwide populationbased prospective cohort study	140425	NR	NR	Asthma	Swedish Hospital Discharge Register	SIR	National Swedish Cancer Registry	1–14	None

Abbreviations: BMI, body mass index; CI, confidence interval; E, exposure; het, heterogeneity; HR, hazard ratio; M, multicenter; NA, not available; NE, non-exposure; OR, odd ratio; S, single center; SIR, standardized incidence ratio; .

Eight of the nine studies involved multicentric data [20–23, 25–28], whereas only one was single center study [24]. Four studies were conducted in North America [20, 22, 24, 27], four in Europe [21, 23, 25, 28] and one in Australia [26]. For study design, eight were population-based cohort study, whereas one was community-based cohort study. The included studies investigated allergies categorized as asthma, hay fever, atopy or other allergic related conditions. The exposure of allergy was ascertained mostly by self-administered questionnaires, while others through research or health care databases, social insurance institution register and hospital discharge register. The following covariates were frequently applied for the adjustment for statistical analyses: age, sex, smoking status, body mass index, alcohol intake and physical activity (Table 1).

### Study quality

Supplementary Table S3 lists the quality scores of these studies based on the Newcastle-Ottawa scale. The methodological quality score were considered high in seven of nine cohort studies [21–23, 24–27], and moderate in four [20, 21, 23, 28]. Most studies had full scores for the representativeness of the exposed and non-exposed cohort, but some lacked scores for comparability on the basis of design or analysis having not controlled for possible confounders. Other studies lacked scores for inadequacy of follow-up.

### Allergies and risk of CRC

**Figure 2:**
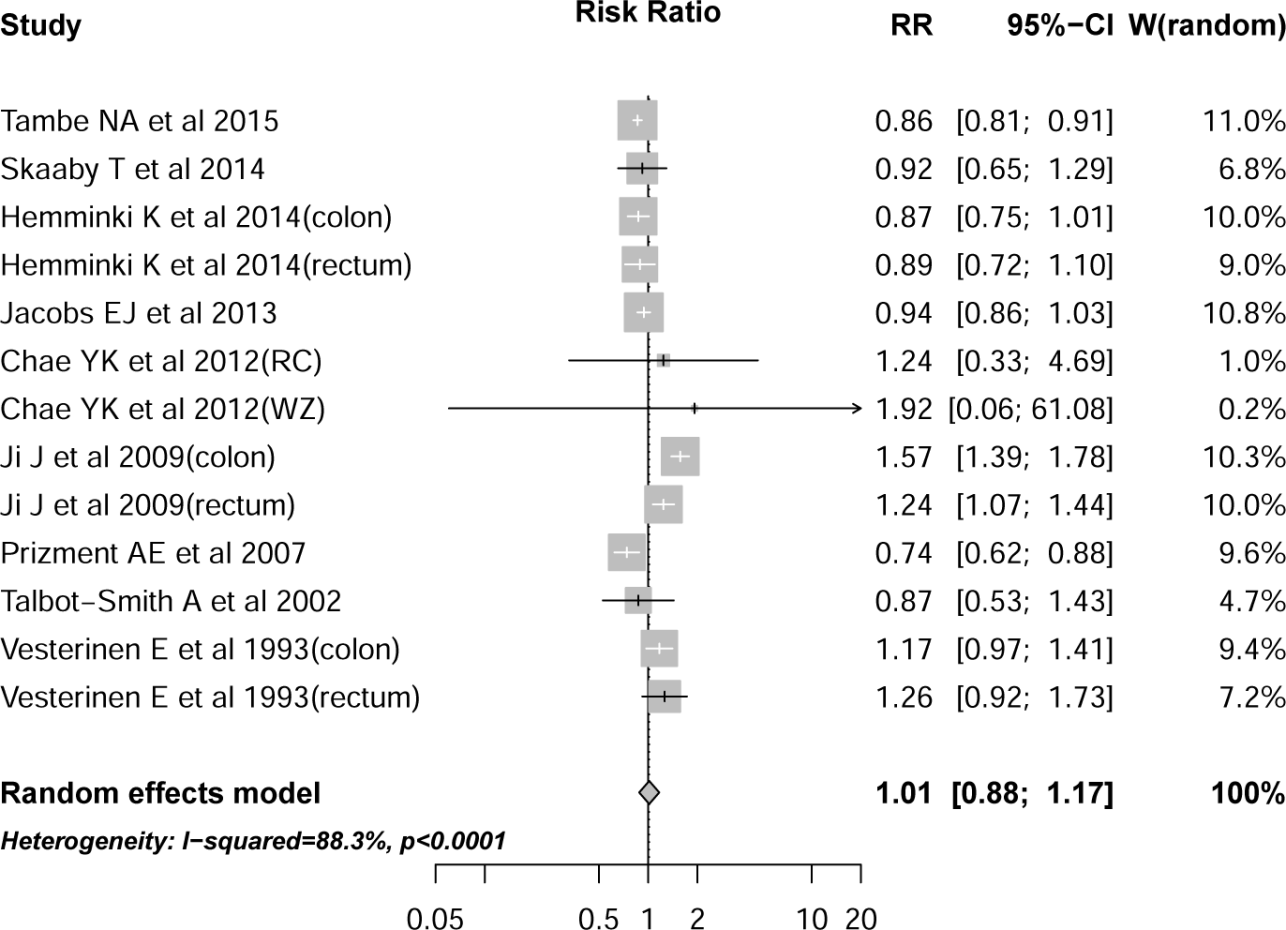
Association between history of allergy and risk of colorectal cancer

Meta-analysis of all cohort studies showed that the history of allergy (as compared to no history of allergy was not associated with a statistically significant reduction in CRC incidence (*n* = 9 studies; adjusted RR 1.01, 95 % CI 0.88 to 1.17) (Figure 2). We selected a random-effects model when performing meta-analyse, and considerable heterogeneity was seen between studies (Cochran ’ s Q test *P* < 0.01, I^2^ = 88.3 % ). Significant reduction in heterogeneity was seen when studies involved in colorectum, hay fever, USA/Canada region, sample size less than 1000, questionnaire based assessment method and quality score > 6, indicating that the heterogeneity could be explained partly by these factors (Table 2).

**Table 2:** Subgroup analyses according to some baseline characteristics

Variables	RR	95%CI	Heterogeneity (%)	*P* for subgroup difference	No. of includedStudies
Total	1.01	0.88 to 1.17	88.3	-	9
Tumor location				0.855	
Colon	1.01	0.81 to 1.25	92.4%		6
Rectum	0.94	0.77 to 1.15	83.8%		6
Colorectum	0.92	0.70 to 1.21	0		3
Sex				0.938	
Male	0.93	0.81 to 1.07	65.1		4
Female	0.94	0.80 to 1.09	65.8		6
Allergy type				0.006	
Asthma	1.16	0.96 to 1.42	77.5		5
Hay fever	0.95	0.85 to 1.06	0		4
Research region				0.112	
USA/Canada	0.89	0.84 to 0.93	1.3		3
Europe	1.04	0.86 to 1.27	88.5		6
Research center				0.003	
Single	0.74	0.62 to 0.88	-		1
Multicenter	1.05	0.90 to 1.22	88.2		8
Sample size				0.686	
≥ 10000	1.02	0.88 to 1.18	91.2		7
< 10000	0.92	0.58 to 1.46	0		2
Exposure assessment method				0.069	
Questionnaire based	0.89	0.81 to 1.01	41.2		6
Health care-based registry	1.13	0.89 to 1.42	90.8		3
Adequate baseline characteristics adjusted				0.069	
Yes	0.89	0.81 to 1.01	41.2		6
No	1.13	0.89 to 1.42	90.8		3
Study quality				0.272	
Quality score > 6	0.95	0.84 to 1.05	58.9		7
Quality score ≤ 6	1.15	0.80 to 1.49	95.0		2

Abbreviations: CI, confidence interval; RR, relative risk.

### Sensitivity analysis and publication bias

Sensitivity analysis suggested that exclusion of any one of the studies in turn did not alter the trend of the summary estimate. Further subgroup analysis stratified by some baseline characteristics showed that the association almost remained constant across the subgroups (Table 2).

**Figure 3:**
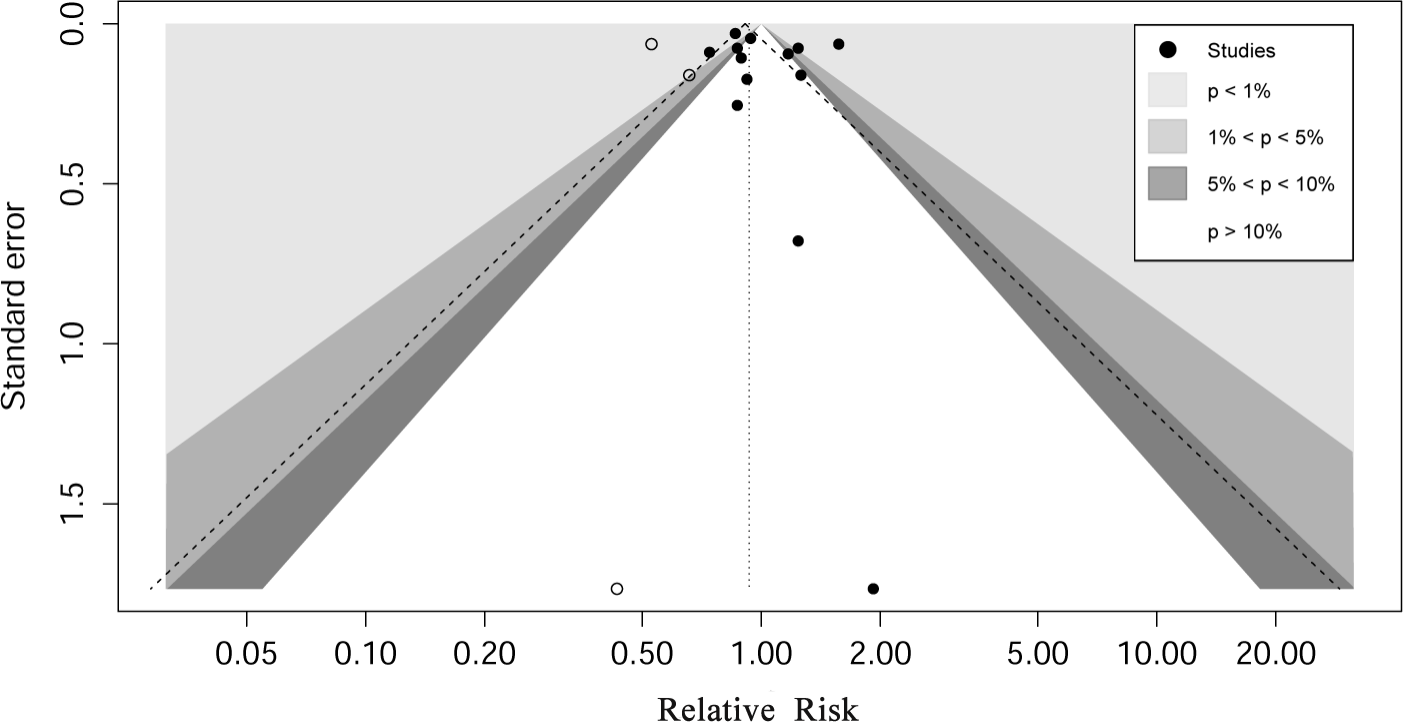
Contour enhanced funnel plot for meta-analysis of the association between history of allergy and risk of colorectal cancer

Visual impression of the funnel plot revealed some asymmetry. However, the Egger ’ s regression asymmetry test (*P* = 0.42) suggested no publication bias. The results of the trim-and-fill method indicated that three studies might have been missing and the adjusted pooled RR was 0.93 (95 % CI 0.79 to 1.09) when inputing these three hypothised studies (Figure 3), which was consistent with the main results.

## DISCUSSION

### Principle findings

In this meta-analysis involving 9 cohort studies analyzing the effect of allergies on modifying the risk of CRC in more than 775,000 individuals, we found that history of allergy was not associated with a decreased risk of CRC. This neutral association was independent of gender, tumor location, research region, allergy exposure assessment method, allergy type, sample size, study quality, or allergy type. Sensitivity analysis and the trim-and-fill method confirmed the main findings.

### Strengths of the study

To the best of our knowledge, this is the first meta-analysis investigating the association between history of allergy and risk of CRC exclusively with the largest sample size till now. The strengths of our study include a contemporaneous and exhaustive search of the major global electronic databases using a comprehensive search strategy, which allow us identify sufficient studies and summary the data from over 775,000 recruited individuals, thus objectively assess the association between ACCs and risk of CRC. Additionally, though limited number of studies were involved in some of the subgroup analyses, most of the studies were large-scale cohorts with a median sample size of 77,952 (range, 3,308 to 199,112) and large sample size implied high statistical power. Second, the literature search, eligibility assessment, data extraction, and quality assessment were conducted independently by at least two investigators and one senior author. Third, in order to more conservatively calculate the risk estimate of CRC, we used a random effects model to combine data in overall population and subgroup analyses were thoroughly undertaken according to gender, tumor location, type of allergy and other baseline characteristics. Fourth, almost all of the studies selected were limited to those with population-based registries with representative samples, which provided better evidence than those provided by data derived from convenience samples. Finally, we assessed for publication bias using different approaches (funnel plot, Egger's test as well as trim and filled method), giving sufficient evidence to confirm the evidence based on the current available studies.

### Limitations of the study

Several limitations should be addressed. First, as a meta-analysis of observational studies, there was a lack of the experimental random allocation of the intervention like randomized controlled trials which was an optimal method to test exposure – outcome hypotheses. Second, the adjusted variables varied among studies. They generally failed to adjust these following important risk factors for CRC in all studies: family history of CRC or other tumors, diet habits, smoking, alcohol use, nonsteroidal antiinflammatory drugs (NSAID) use and other related factors, which could be some of the influential factors of CRC. Moreover, three studies used SIRs to estimate CRC risk [21, 23, 28]. SIRs, known to inherently correspond to RR estimates and obtained only through adjustment for age and calendar time [29], would likely to overestimate cancer risk [30, 31]. Third, the summary RR must be interpreted with caution as the ascertainment of allery exposure varied among included studies. Most of the studies involve self-administered questionnaire to report allergic conditions and symptoms, while some used hospital discharge register. Considerable inter-study heterogeneity did exist but we made an attempt to account for this variation by conducting subgroup analyses and some of the examined variables were really attributed to the significant heterogeneity (Table 2). In addition, as the included studies were all study-level studies, we could not abstract more detailed information of each individual, thus some of the subgroup analyses we were interested in could not be performed. For example, for some specific allergy types, though we have investigated asthma and hay fever subtype, limited number of studies in each subtype resulted in insufficient statistical power to draw definite conclusions for the true associations. Another major limitation was that the assessment method of exposure was mostly through self-administered questionnaires rather than the objective laboratory measurement of allergy. So we propose that more well designed large-scale cohort studies investigating the association between allergies and CRC risk should be aunched. Furthermore, funnel plot asymmetry indicated the overestimation of the effect size. However, the adjusted RR by using trim and filled method did not largely alter the statistical significance of the results, indicating the robustness of our findings. Finally, we did not include the unpublished studies. Since the omission the unpublished studies will lead to asymmetry of the funnel plot, the combined effect from meta-analysis will overestimate the effect of exposure. Such asymmetry might also result from the overestimation of the effects of exposure in smaller studies of lower methodological quality [32].

The mechanisms of tumorigenesis associated with allery are controversial. There have been two contradictory theories proposed. One theory was that cancer risk could be reduced by some allergic conditions through immune surveillance, inducing immune reactions to remove malignant tumor cells, whereas the other theory proposes that allergic conditions can result in continuous tissue inflammation, damage and repair, which increases the risk of cancer [33, 34]. A meta-analysis by Olson et al. supported the former theory in pancreatic cancer that allergic conditions could be a protective factor for specific cancer risk. However, they only found the reduced risk for hay fever and allergy to animals, but not for asthma or other allergies [35]. Our study finds that neither hay fever nor asthma has a protective association. Perhaps some larger population-based prospective cohort studies can give further evidence for this association and provide directions for future research on this topic.

In summary, this systematic review and meta-analysis provide evidence that do not support a substantial a protective or harmful association between history of allergy and risk of CRC. Future well-designed prospective cohort studies should be conducted to better understand this association. Furthermore, studies should include analysis on more types of allergy in order to investigate if the effect of specific type of allergy on the tumorigenesis of CRC.

## MATERIALS AND METHODS

### Search strategy

A systematic search of literature was performed on January 26, 2016, in PubMed and Embase from the initial available date according to the Cochrane review guidelines. We used the following sets of Mesh/Emtree terms for searching: allergy, asthma, allergens and allergic rhinitis; colorectal neoplasms, colonic neoplasms and rectal neoplasms. The detailed search strategies are presented in Supplementary Tables S1–S2. Besides, we also conducted manual search of the reference lists of related reviews or meta-analyses identified through the above systematic database searches. We also searched the titles of published papers in the following major surgery-related journals for the period of last 10 years: Annals of Surgery, British Journal of Surgery, JAMA Surgery, Annals of Surgical Oncology, Surgery, and American Journal of Surgery. However, we did not include ‘grey’ or unpublished literature in our meta-analysis.

### Eligibility criteria

Allergies or allergic conditions were defined as a self-report history of having been previously diagnosed by a physician that the respondent had asthma, hay fever, skin or food allergy, or any other allergy. All abstracts examining risk estimates of the association between history of allergy and CRC were screened for full-text review. Studies were considered eligible for inclusion if they satisfied the following criteria: prospective or retrospecitve human cohort studies; studies investigating the association between history of allergy and CRC and reporting the corresponding RRs or SIRs of CRC or sufficient data to calculate them.

### Data extraction and study quality assessment

Two reviewers (J.Y. and A.T.) independently evaluated each eligible study and extract related data. Disagreements were resolved by discussion or by a third reviewer (H.Z.). A predesigned standardized data collection form regarding the baseline characteristics including the following items was used: first author, publication year, research country, inclusion period, study name, center involved, study design, sample size, mean/median age of included individuals, percent of male individual, type of allergy, exposure assessment method, measure of associations, outcome assessment, years of follow-up and adjusted variables.

We used the Newcastle–Ottawa Scale (NOS) [36] to assess study quality, which was developed to give a full assessment of the methodological quality of observational studies. Eight items totally 9 points across three major scales are judged including selection of the participants, comparability of the participants and outcomes. Two reviewers (Q.Z, L.M) scored each study and each study receives an overall score for methodological quality, with a score of > 6 (totally 9) indicating low risk of bias and a score of ≤ 6 suggesting high risk of bias.

### Statistical analyses

The meta-analysis was performed abided by the preferred reporting items for systematic reviews and meta-analyses (PRISMA) statement [37]. All statistical analyses were conducted using Stata software (StataCorp. 2013; Stata Statistical Software: Release 13, College Station, TX: StataCorp LP).

We extracted the adjusted relative risks (RRs), odd ratios (ORs), hazard ratios (HRs), standardized incidence ratios (SIRs) and corresponding 95% confidence intervals (CIs), if available, from the included studies which were used to assess the association between history of allergy and CRC risk. For the low absolute risk of CRC, it is expected that the four measures of association can yield similar estimates of RR. Therefore, we can reasonably consider that the pooling of RR estimates can ensure the comprehensiveness of the meta-analysis and maximization of the statistical power [38, 39].

Random-effects models were used to evaluate the pooled RR for the association between history of allergy and CRC incidence [40] because results from the random-effects model could more conservatively presented the true underlying effect among the included studies with varied backgroud. Between-study heterogeneity was evaluated by the chi-square test and I^2^ statistic [41]. An I^2^ > 50% was considered significant heterogeneity.

Potential publication bias was evaluated through visual inspection of funnel plots combined with the Egger's regression test [42] served as statistical assessment of publication bias. We also conducted the Duval and Tweedie nonparametric “trim and fill” approach to further assess the impact of publication bias on the combined estimate [43]. Subgroup analysis was also performed according to some of the baseline characteristics regarding population features (sex, tumor site and research region) , exposure (allergy exposure assessment method and allergy type), study design (research center and sample size) and other related factors which some of the heterogeneity may potentially attribute to. All statistical tests were two-sided, and significance was defined as a *P* value less than 0.05.

## SUPPLEMENTARY MATERIALS FIGURES AND TABLES





## Abbreviations

CRC: Colorectal cancer
RR: relative risk
CI: Confidence interval
NOS: Newcastle–Ottawa Scale
PRISMA: Preferred reporting items for systematic reviews and meta-analyses
ORs: Odd ratios
HRs: Hazard ratios
SIRs: Standardized incidence ratios
