# Supplementary material for: Allergies and risk of colorectal cancer: a systematic review and meta-analysis of observational studies
Source: Oncotarget. 2017 Jan 11;8(9):14646–54. doi: 10.18632/oncotarget.14599 (PMC5362432; doi:10.18632/oncotarget.14599)
Supplement: Supplementary file 1 [file oncotarget-08-14646-s001.pdf]

Allergies and risk of colorectal cancer: a systematic review and meta-analysis of observational studies

Supplementary Materials

Supplementary Table S1: Detailed search strategy for pubmed

1. "Allergy and Immunology" [Mesh]
2. "Asthma" [Mesh]
3. "Allergens" [Mesh]
4. "Rhinitis, Allergic, Seasonal" [Mesh]
5. (Allerg* OR Hypersensitivit* OR Asthma* OR atopy OR ‘hay fever’ OR hayfever) [Title/Abstract]
6. 1 OR 2 OR 3 OR 4 OR 5
7. "Colorectal Neoplasms" [Mesh]
8. "Colonic Neoplasms" [Mesh]
9. "Rectal Neoplasms" [Mesh]
10. (((colorect*[Title/Abstract]) OR colon*[Title/Abstract]) OR rectum [Title/Abstract]) OR rectal [Title/Abstract]
11. ((((((cancer*[Title/Abstract]) OR tumor*[Title/Abstract]) OR tumour*[Title/Abstract]) OR carcinom*[Title/Abstract]) OR neoplas*[Title/Abstract]) OR adenocarcinoma*[Title/Abstract]) OR malignan*[Title/Abstract]
12. 10 AND 11
13. 7 OR 8 OR 9 OR 12
14. 6 AND 13

Supplementary Table S2: Detailed search strategy for embase

1. "allergy'/exp
2. 'atopy'/exp
3. 'pollen allergy'/exp
4. 1 OR 2 OR 3
5. (Allerg* OR Hypersensitivit* OR Asthma* OR atopy OR ‘hay fever’ OR hayfever) :ab,ti
6. 4 OR 5
7. 'colorectal cancer'/exp
8. 'colon cancer'/exp
9. 'rectum cancer'/exp
10. ((colorect* OR colon* OR rectum OR rectal) and (cancer* OR tumor* OR tumour* OR carcinom* OR neoplas* OR adenocarcinoma* OR malignan*)):ab,ti
11. 7 OR 8 OR 9 OR 10
12. 6 AND 11

Supplementarey Table S3: Methodological quality of included studies based on the Newcastle–Ottawa scale for cohort studies

Observational studies[a]					
**Study**	**Design**	**Selection**	**Comparability**	**Outcome/exposure**	**Overall quality (max 9)**
**Tambe NA et al. 2015**	Cohort	★★★	★★	★★	7
**Hemminki K et al. 2014**	Cohort	★★★★		★★	6
**Skaaby T et al. 2014**	Cohort	★★★★	★	★★	7
**Jacobs EJ et al. 2013**	Cohort	★★★	★★	★★★	8
**Chae YK et al. 2012**	Cohort	★★★	★★	★★	7
**Ji J et al. 2009**	Cohort	★★★★		★★	6
**Prizment AE et al. 2007**	Cohort	★★	★★	★★★	7
**Talbot-Smith A et al. 2002**	Cohort	★★★	★	★★★	7
**Vesterinen E et al. 1993**	Cohort	★★★★	★	★★	7

[a]Study quality assessment of observational studies performed using the Newcastle–Ottawa scale (each asterisk represents if individual criterion within the subsection were fulfilled).

Supplementarey Table S4: PRISMA 2009 Checklist. See Supplementary_Table_S4
